# Recruiting families for an intervention study to prevent second-hand smoke exposure in children

**DOI:** 10.1186/s12887-018-0983-4

**Published:** 2018-01-31

**Authors:** Sasha G. Hutchinson, Constant P. van Schayck, Jean W. M. Muris, Frans J. M. Feron, Edward Dompeling

**Affiliations:** 1 Department of Paediatric Respiratory Medicine, Maastricht University Medical Centre (MUMC+) / CAPHRI School for Public Health and Primary Care, P.O. Box 616, 6200 MD Maastricht, the Netherlands; 2 Department of Family Medicine, MUMC+ / CAPHRI, P.O. Box 616, 6200 MD Maastricht, the Netherlands; 3 Department of Social Medicine, MUMC+ / CAPHRI, P.O. Box 616, 6200 MD Maastricht, the Netherlands

**Keywords:** Hard-to-reach populations, Second-hand smoke, Children, Recruitment

## Abstract

**Background:**

We evaluated the effectiveness of different recruitment strategies used in a study aimed at eliminating/reducing second-hand smoke (SHS) exposure in Dutch children 0–13 years of age with a high risk of asthma.

**Methods:**

The different strategies include: 1) questionnaires distributed via home addresses, physicians or schools of the children; 2) cohorts from other paediatric studies; 3) physicians working in the paediatric field (family physicians, paediatricians and Youth Health Care (YHC) physicians); and 4) advertisements in a local newsletter, at child-care facilities, and day-care centres.

**Results:**

More than 42,782 families were approached to take part in the screening of which 3663 could be assessed for eligibility. Of these responders, 196 families met the inclusion criteria for the study. However, only 58 (one third) could be randomised in the trial, mainly because of no interest or time of the parents. The results showed that recruiting families who expose their children to SHS exposure is very challenging, which may be explained by lack of ‘recognition’ or awareness that SHS occurs in homes. The presence of asthma in the family, respiratory symptoms in the children, and even incentives did not increase parental motivation for participation in the study.

**Conclusions:**

The recruitment process for an intervention program addressing SHS exposure in children was considerably more challenging and time consuming than anticipated. Barriers at both a parents level and a doctor’s level can be discriminated.

**Electronic supplementary material:**

The online version of this article (10.1186/s12887-018-0983-4) contains supplementary material, which is available to authorized users.

## Background

Second-hand smoke (SHS) exposure in children is still a problem of major societal concern. The WHO estimates that about 40% of children worldwide are exposed [1]. SHS exposure in Dutch children may have decreased over the past years but not in families of low social economic groups [2]. The adverse health effects in terms of associated morbidity and mortality of SHS exposure in children are large [3], including increased risk of sudden infant death syndrome, respiratory tract infections and complaints, asthma, and even meningitis in children [4–7]. Therefore, effective strategies are urgently needed to prevent SHS exposure in children [8]. We developed a motivational interviewing intervention with feedback of the children’s urine cotinine level to prevent SHS exposure in children with a high risk of asthma (the PREPASE study [9]). The purpose of the PREPASE study was to test the effectiveness of the intervention program in a randomized controlled trial (RCT) with approximately 150 families with children 0–13 years of age with a high risk of asthma (due to asthma in a biological parent and/or sibling) and SHS exposure at home.

The aims of this study were: 1) to describe and evaluate the different recruitment strategies of the PREPASE study; 2) to assess the influence of socioeconomic factors, presence of asthma in the family, and respiratory symptoms in the children on the effectiveness of the recruitment process.

## Methods

**Fig. 1:**
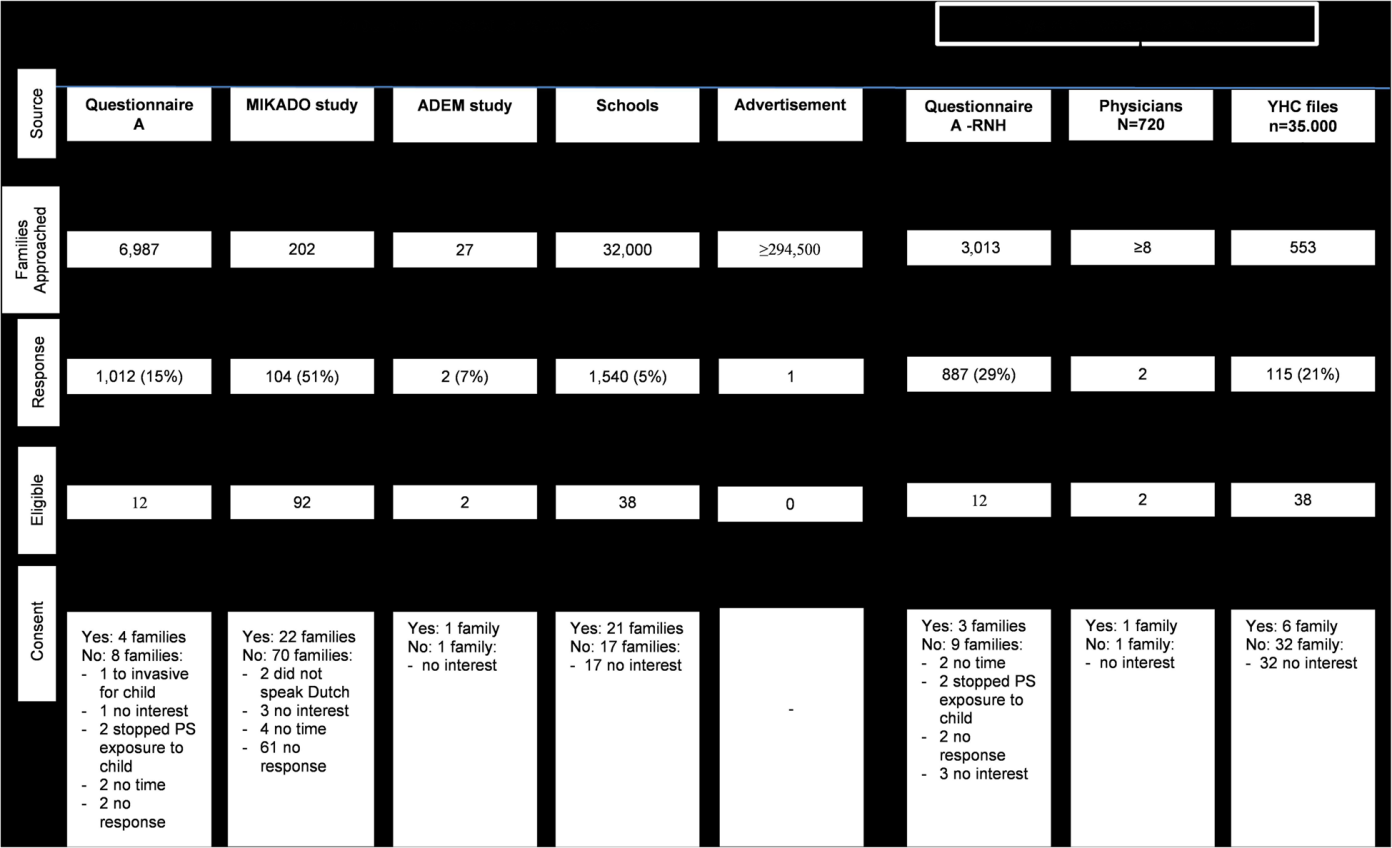
Flowchart recruitment and enrolment

The study protocol and recruitment strategies for the PREPASE study were previously described [9]. The recruitment strategies are summarized in Fig. 1.

### Population-based strategies

#### Survey among 6,987 families with children aged 0–13 years

Our initial strategy was to select eligible candidates for the PREPASE study via a survey. The civil affairs departments of three communities (Heerlen, Maastricht and Sittard-Geleen) in South-Limburg, Netherlands, provided 22,532 addresses where children within the age range of 0–13 years were living. We randomly selected 6,987 of those addresses and sent a package including an invitation letter to participate in the study, an informed consent form, two questionnaires (responder (A) and non-responder (B), see Additional file 1) and a return envelope. Parents were informed that participation was voluntary, and that every 50th participant would receive a gift voucher of €25. Non-responders would receive a single reminder letter after two weeks. Parents who wanted to participate could complete a questionnaire A consisting of 91 items including; family characteristics (nine questions), the child’s general and respiratory health (including the ISAAC questionnaire [8], 38 questions), parental smoking behaviour and SHS exposure to the child (44 questions of which 36 were double questions 18 for the primary responder and 18 for their possible partner). Parents who were not willing to participate with the survey were asked if they could complete questionnaire B (11 items, including gender and birth date of the child, relationship of the caregiver(s) to the child, wheezing and respiratory complaints in the past 12 months, SHS exposure and reasons for nonparticipation) for the purpose of a non-responder analysis. Parents could give permission to be contacted for a possible follow-up study via the informed consent form. Eligible families who gave permission were telephoned and provided with information about the RCT. Interested parents received an information letter of three pages describing the aim of the study (to test the effectiveness of a program to prevent SHS in children), study duration, measurements, pros (gaining more knowledge of the respiratory health of their child) and cons (time investment) of participation, protection of their data, incentive of €100 for complete participation, and a gift for the child after every lung function measurement, and further instructions. The letter also included an informed consent form and return envelope.

#### Participants from other paediatric studies

Participants were selected from the MIKADO [10] and the ADEM study [11] from the department of paediatric respiratory medicine Maastricht University Medical Centre (MUMC+). The MIKADO study was recruiting families during the same time as the PREPASE study, by means of an electronic survey (64 items) about obesity and asthma among 40,000 families with children 6–16 years of age living in South-Limburg. Families that were not eligible for the MIKADO study or who completed the ADEM study, and in both cases were eligible to participate in the PREPASE study and gave permission to be contacted for possible follow-up studies, received hardcopy information concerning the PREPASE intervention study as mentioned above. Parental willingness for participation was assessed two weeks later by phone. Parents who wanted to participate received an informed consent form and return envelope and were randomised in the study.

#### Schools

Parents of 32,000 children aged 6–12 years received an invitation letter to participate in an electronic survey study (see Additional file 1) through the primary school of their child. Recruitment on primary schools in the Netherlands was feasible in a similar study [12]. The 9 main school organizations in South-Limburg were informed about the aims of the PREPASE study and asked for permission to contact their schools. Seven organisations gave permission. First, 205 schools were informed about the study via e-mail and were telephoned one week later to ask for their participation. Sixty-four per cent (*n* = 132) agreed to distribute letters to the children of their schools. The letters were personally delivered to the schools by a member of our research team. All children received a letter addressed to their parents asking them to participate in an electronic survey about the respiratory health of children living in South-Limburg. Each letter contained an original log-in name and password. To encourage participation, parents were informed that we would draw 50 amusement park tickets at the end of the study. The survey consisted of 44 items; general characteristics of the child and family (10 questions), the child’s general and respiratory health (23 questions) and SHS exposure to the child (4 questions for the primary responder and 4 for their possible partner), and questions regarding participation in the follow-up study (3 questions). At the end of the questionnaire, brief information of the PREPASE study was given in 6 short sentences. Parents were informed that they were possibly eligible to participate in the PREPASE study in case they smoked in the presence of their child at home and the child had a first degree relative with asthma. Furthermore, parents also received information about our aim (to help us make a program to reduce SHS exposure in children at high risk for asthma), information on study duration and non-invasiveness of the measurement, and that the child would receive a gift after every measurement and families an incentive of €200 after complete participation in the trial. Additionally, parents were asked which strategy or intervention program they thought was better to educate parents about SHS exposure in children and how to prevent it. All parents received a reminder letter via the primary schools of their children after two weeks.

#### Advertisements

A single advertisement was placed in a local newsletter that is freely distributed to almost every house in South-Limburg (*n* = 294,500) once per week. Additionally, advertisements were placed in most (444) child-care facilities and day-care centres in South-Limburg.

### Physician based strategies

#### Survey among 3,013 families with children aged 0–13 years

The Registration Network of Family Practices (RNH Dutch acronym), uses a computerised database containing patient characteristics of 21 primary care physicians’ group practices in Limburg, the Netherlands [13]. Nine out of the 21 primary care physicians’ group practices of the RNH agreed to participate in the study. The main reason for not participating was lack of time. The participating physicians selected all children 0–13 year of age from the RNH database (*n* = 3,013) and provided them with a personalised information package (the same information package as the 6,987 families mentioned above under population based strategy 1 and during the same time period. Non-responders received a reminder letter from their physicians after two weeks.

#### Physicians working in the paediatric field

All primary care physicians, paediatricians and youth health care (YHC) physicians (*n* = 720) in South-Limburg were asked to select and invite eligible families to participate from their patient registries or actively during consultations (during the year 2011). The physicians were informed about the study in various ways to encourage their participation: via their newsletters and e-mail addresses, telephone calls to their practices and by giving oral presentations about the PREPASE study. To further encourage the primary care physicians, we agreed to give them an incentive of €25 per selected eligible family and additionally €75 for every family they personally invited for the study whom also chose to participate in the study.

#### Patient registries

Registries of 35,000 children in South-Limburg at the Regional Public Health Service department of YHC were checked for eligibility (child 0–13 years of age with high risk of asthma and SHS exposure at home). The YHC is a preventive health care for all children aged 0–19 years living in the Netherlands purposed to promote, protect and monitor the physical, psychological, social and cognitive development of children and advise parents and children about healthy development for the child [14]. In 2009, 93% of all children aged 0–4 years were reached by the YHC [15]. Therefore, the YHC service is an opportune place to support recruitment of children in prevention studies. All available files were checked by two research assistants. Eligible families received an invitation letter from their physician to participate in the study. We also tried to contact the families by phone. The families were informed that the MUMC+ was doing a study about irritable substances and respiratory health in children. Families were asked to complete an answering form stating if they wanted to be contacted (yes or no) by a member of the research group for more information. Interested families were offered a lung function measurement and urine cotinine analysis of their children at home. During the measurement parents were informed about irritable substances that can cause respiratory complaint of children, one of which is SHS exposure in children. The parents were informed about the PREPASE study and asked if they wanted to participate.

### Data analysis

Data were analysed using SPSS version 20 (SPSS INC., Chicago, Il, USA). The descriptive statistics of the recruitment strategies: questionnaire A, MIKADO and schools are presented. Data from *N* = 154 children (obtained via questionnaire A, MIKADO and schools) who were eligible to participate in the RCT were combined to check for possible difference between the families who provided consent to participate in the study and those who did not. Chi-square tests and logistic regression analysis were used for the categorical variables and the independent t-test for the continuous variables. Additionally, parents’ choice for the most effective strategy to educate parents about SHS exposure in children and how to prevent it was analysed with multinomial regression analysis.

## Results

### Recruitment and enrolment

A summary of the recruitment strategies is shown in Fig. 1:

1. *Population based strategies:* at least 39,216 families were approached (excluding the strategy approach via advertisements). The combined response rate was only 7% (*n* = 2,667). The response rate from the MIKADO group was substantially higher than the other strategies probably because the families were approached by letter and a phone call. The telephone numbers of the participants of the other strategies were not available. Just 6% (*n* = 151) of all the responders of the population based strategies were eligible to participate in the RCT and about one-third of those (*n* = 49; 32%) provided consent to be randomized in the study. The main reason for not participating in the RCT was due to lack of interest. Sixty per cent of the eligible families did not respond to our invitation letter and or phone calls. Furthermore, based on the selection of MIKADO data all participants were eligible for the PREPASE study. However, during the telephone contact 12 families were no longer eligible because the parent reported they had stopped smoking or had never smoked in the first place.
2. *Physician based strategies:* 720 physicians were asked to help recruit participants for the PREPASE study and 35,000 patient registries at the YHC department of the Regional Public Health Services in South-Limburg were checked for eligibility. In total, at least 3,566 families were approached via the physician based strategies. The mean response rate was 28% (*n* = 1,010). In total, 7% (*n* = 70) of all the responders were eligible to participate in the RCT. A few as 10% (n = 7) gave consent for the RCT. The main reason for not participating in the trial was no interest. The total amount of families approached via their physicians is unknown. One primary care physician communicated back with us about inviting two eligible families, two paediatricians provided contact information of five families that were interested, and one YHC physician provided information of one family that was interested to participate in the PREPASE study.

### Characteristics of the responders and non-responders

The characteristics of the responders of questionnaire A, MIKADO and the electronic survey via the schools are provided in Table 1. Parental active smoking and SHS in children were rather similar in all four strategies, including the frequencies of the reported respiratory complaints in children. An interesting observation was that a relatively low amount of families reported to have low education. Furthermore, regarding questionnaire A, the response rate of the group approached via physicians (29%) was significantly (*p* < 0.01) higher compared to the group that randomly received the invitation package via their postal address (15%). In total, 5% (*n* = 508) of all the parents that were invited to complete questionnaire A (via communities as well as via physicians (*n* = 10,000)) completed the non-responders questionnaire B. There were no differences between the responders of questionnaire A and questionnaire B with regards to the prevalence of SHS exposure in children, respiratory tract infections in the last 12 months, recent wheezing and asthma (results presented elsewhere [16]), which suggested no selection bias. About 60% reported they did not complete questionnaire A because they did not allow smoking inside their homes, and 50% because their children did not have respiratory complaints. Reasons such as no interest or lack of time were reported less frequently.

**Table 1:** Participants’ characteristics per strategy

Strategy	Questionnaire A communities *n* = 6,987	Questionnaire A physicians *n* = 3,013	MIKADO *n* = 202	Schools *n* = 32,000	Total 42,202
Response (n (%))	1,012 (15)	887 (29)	104 (51)	1,540 (5)	3543 (8)
Primary caregiver responder:					
- Mother (n (%))	843 (83)	757 (85)	87 (84)	1,244 (81)	2,931 (83)
- Father (n (%))	158 (16)	120 (14)	14 (14)	271 (18)	563 (16)
- Other (n (%))	11 (1)	10 (1)	3 (3)	25 (2)	49 (1)
Age of children (mean (SD))	6.2 (4.1)	7.6 (4.2)	9.2 (2.3)	8.1 (2.5)	7.5 (3.6)
Age of mother (mean (SD))	38.9 (6.3)	40.3 (5.6)	Not asked	39.8 (5.1)	39.8 (5.7)^c^
Highest parental education^a^					
- Low (n (%))	31 (3)	41 (5)	23 (22)	41 (3)	136 (4)
- Middle (n (%))	268 (27)	262 (30)	50 (48)	405 (29)	985 (28)
- High (n (%))	404 (40)	454 (51)	28 (27)	730 (45)	1,616 (46)
- Academic (n (%))	300 (30)	126 (14)	3 (3)	332 (22)	761 (21)
- Other (n (%))	0	0		32 (2)	32 (1)
- Missing (n (%))	9 (1)	4 (1)			13 (0)
Active smoking of both parents (n (%))^b^	306 (16)	257 (15)	141 (70)	507 (17)	1,211 (34)
Active smoking at least one parent per family (n (%))	250 (25)	210 (24)	104 (100)	390 (25)	954 (27)
Smoking in the presence of the child (n (%))	131 (13)	135 (15)	104 (100)	141 (9)	511 (14)
Wheezing ever (n (%))	283 (28)	255 (29)	29 (28)	478 (31)	1,045 (29)
Wheezing in the past 12 months (n (%))	144 (14)	114 (13)	7 (7)	229 (15)	494 (14)
Asthma diagnosis (n (%))^c^	66 (13)	65 (12)	0	161 (11)	292 (8)
Respiratory tract infection in the past 12 months (n (%))	413 (41)	278 (31)	Not asked	567 (37)	1,258 (37)^d^
Asthma in the first degree relative (n (%))	258 (26)	215 (24)	104 (100)	423 (28)	1,000 (28)

^a^ Parental education definition: Low: range no education to lower vocational education; Middle: range general secondary education to middle vocational education; High: range higher general secondary education to high vocational education; Academic: university education. ^b^ prevalence calculated from the total amount of parents individually in each group (Questionnaire A community: 1925 parents, Questionnaire A physicians: 1718 parents, MIKADO: 202 parents, Schools: 2929 parents); ^c^ only children 6 years and older included (questionnaire A community: 515 children, Questionnaire A physicians: 557 children; ^d^ prevalence calculated from *n* = 3439 due to exclusion of *n* = 104 children form the MIKADO group

Regarding the electronic survey via schools, 42% (*n* = 649) of parents reported that they were willing to help the PREPASE study develop a program to stop SHS exposure in children. Twelve per cent (*n* = 76) of these parents also reported SHS exposure in their children at home. Among the parents that were not willing to help the PREPASE study, 78% reported that they did not want to participate because they did not allow smoking in their homes and 54% reported that their reason was because their child did not have respiratory complaints. No interest (6%) or lack of time (6%) was less frequently reported as reasons for not wanting to participate in the study. Furthermore, the parents were asked what strategy they would find more effective for preventing SHS in children (Table 2). In general, 44% of all parents found that SHS exposure in children could best be prevented by a motivational interviewing intervention program. Compared to parents not exposing their children, parents exposing their children to SHS were less inclined to indicate motivational interviewing and a group program in their neighbourhood as an effective strategy for prevention of SHS exposure. Almost, one third of the parents with SHS exposure in their children reported that an internet intervention program would be more effective. Last, data of 154 eligible participants were combined, see Table 3. We found no difference between the group that participated and the group that did not participate in the RCT.

**Table 2:** Parents’ choice for most effective strategy to inform parents about passive smoking in children and how to prevent it

Strategy	Smoking in the presence of the child		Total (*n* = 1,540)	OR (95% CI)^a^
	YES-*n* = 141 (9)	NO-*n* = 1,399 (91)		
An internet program (n (%))	45 (32)	131 (9)	176 (11)	2.0 (1.2–3.4)^#^
A program via telephone contacts (n (%))	3 (2)	11 (1)	14 (1)	1.6 (0.4–6.1)
A program consisting of motivational interviewing with a trained coach at home (n (%))	25 (18)	655 (47)	680 (44)	0.2 (0.1–0.4)^#^
A group program for parents at a central location in a neighbourhood (n (%))	13 (9)	161 (12)	174 (11)	0.5 (0.2–0.9)^#^
A combined program consisting of contacts by phone and motivational interviewing at home (n (%))	29 (29)	290 (21)	319 (21)	0.6 (0.3–1.0)
Other (n (%)) - Do not know (60 (34%)) - TV documentaries and confrontational tv-advertisements (29 (16%)) - Stop active smoking (21 (12%)) - Smoking ban / take tobacco products of the market (18 (10%)) - Education about smoking at schools (12 (7%)) - Direct confrontation during e.g. doctor visits (11 (6%)) - Combination of all programs (9 (5%)) - Increase taxes and prices of tobacco products (5 (3%)) - Make SHS exposure in children punishable by law (5 (3%)) - Increase health insurance of smokers (3 (2%)) - Tailored to personal needs and intervention strategy (2 (1%)) - Free smoking-cessation programs or therapy (1 (1%)) - Nothing will help (1 (1%))	26 (19)	151 (11)	177 (12)	reference

^a^ OR = Odds Ratio, 95%CI = 95% Confidence Interval

^#^*p* < 0.05

**Table 3:** Comparison between eligible participants (n = 154) who participated and who did not participate in the randomized controlled trial (RCT)

	RCT -YES n = 50	RCT- N0 n = 104	OR (95% CI)^a^	*p*-value
Age of the child (mean (SD))	8.2 (2.4)	9.2 (2.5)		*p* = 0.97
Wheezing ever (n (%))	17 (34)	33 (32)	1.1 (0.5–2.3)	*p* = 0.78
Wheezing last 12 months (n (%))	7 (14)	12 (12)	–	*p* = 0.28
SHS by^#^:				
Mother (n (%))	23 (46)	31 (30)	Reference	
Father (n (%))	12 (24)	36 (35)	2.2 (1.0–5.2)	*p* = 0.06
Both parents (n (%))	15 (30)	36 (35)	1.8 (0.8–4.0)	*p* = 0.16
Highest parental education^#^:				
Low (n (%))	9 (18)	14 (13)	Reference	
Middle (n (%))	24 (48)	48 (46)	1.3(0.5–3.4)	*p* = 0.61
High/Academic (n (%))	16 (32)	41 (39)	1.7 (0.6–4.6)	*p* = 0.34

^a^ OR = Odds Ratio, 95%CI = 95% Confidence Interval

^#^ Values may not add up due to missing values

## Discussion

We described the recruitment strategies of the PREPASE study. The recruitment process was considerably more challenging and time consuming than anticipated, and none of the strategies proved to be very effective. At least 42,782 families were asked to participate. Finally, 196 families met the inclusion criteria for the study but only 58 families participated in the RCT. The observations suggest that we were dealing with at least two main areas of barriers arising from 1) the physicians and 2) the study population.

### Barrier 1: The physicians

The current study showed that even physicians, who play a crucial role in the prevention of SHS exposure in children, were not actively engaged in the study. This is very disappointing, because prevention of SHS exposure in children can decrease morbidity and mortality in children considerably [3]. In previous studies from our department, recruitment of children for research by means of the RNH has shown to be more effective, likely due to the different nature of their research questions [11, 17]. In the PREPASE study, only 9 out of 21 RNH practices participated in the recruitment process. The response rates for questionnaires A were significantly higher in the group that received an invitation via their physicians than an invitation of the project group, suggesting that parents might be more willing to participate when they are approached via their physicians instead of via other strategies [18]. A study evaluating the recruitment of participants via Dutch physicians found that recruitment was more successful when physicians were asked to recruit prevalent cases instead of incident cases, if physicians did not have to be alert during consultation, and when participants were invited by mail. In the PREPASE study, physicians were asked to recruit participants actively during consultations as well as retrospectively through the patient files [19]. But only 4 of 720 physicians reported back to the research group concerning possible participants. So, even the recruitment of “prevalent cases” was not effective. Possibly because not all physicians keep a good record of whether or not a child is exposed to SHS [20], making it difficult for them to identify the eligible families for the study via their patient registries or International Classification of Primary Care (ICPC) registration of nicotine abuses in RNH practices. Comparable recruitment problems in a primary care setting had been described in the recent REFRESH study, assessing the influence of an MI intervention combined with feedback about home air quality on SHS exposure in preschool children [21].

Additionally, in the case of YHC physicians, currently more of their tasks regarding screening and prevention of health problems in children are done by the YHC nurses. Therefore, the YHC nurses were also requested to help to recruit participants for the study, but they did not provide eligible participants either. Nevertheless, physicians working in child-care settings have a unique opportunity to counsel parents for SHS exposure in children but infrequently do so due to barriers such as lack of time, competing priorities and fear of negative parental response [22–24]. A study indicated that physicians are less likely to aid to smoking cessation in socially and economically deprived groups [25]. For instance, one primary care physician reported to our project team that he did not want to participate especially because the families that exposed their children to SHS were of low socioeconomic status, and he did not want to damage the doctor-patient relationship. Similarly, another physician did not want to participate because of the resistance he gets from patients when talking about exposure in children. Other physicians said that they were not able to participate because of lack of time or due to participation in other research. But the majority of physicians was not actively involved and did not provide reasons for non-participation.

### Barrier 2: The study population

In general, the presence of asthma in the family or of respiratory symptoms in the children did not increase parental motivation for participation in the study, neither did the offering of incentives. At the time the PREPASE study started (2010), the Dutch ban on smoking in public places was already in effect. Although the reported prevalence of SHS in Dutch children decreased further since the smoking ban, a considerable amount of children were still exposed to SHS, especially in the group of low-social economic status and heavy smokers [2]. The media attention and social pressure since the smoking ban in public places might have motivated parents to participate in a study like PREPASE in order to receive support to stop SHS exposure in their children. In contrast, the media attention could have also made parents more reluctant to participate in the study due to perhaps feelings of guilt, shame or fear of being criticized. In our experience the last may have played a greater role. Stigmatisation of smokers is a well-known phenomenon in the literature [26]. Some parents reacted very aggressively towards our project team during the telephone contacts regarding SHS in children and others have reported in questionnaires that they were not smokers, yet the questionnaires had the odour of tobacco. Furthermore, the home was probably for parents who did not want to quit smoking their ‘only safe haven’, where they were not waiting for outsiders to educate them about smoking in their own house. Probably, this was also the reason why significantly more parents who exposed their children to SHS reported to find an internet program effective for parental education and prevention of SHS in children, compared to the parents of children without SHS exposure. Moreover, motivational interviewing at home and a group program in the neighbourhood were less mentioned as effective measures against SHS in these parents compared to the parents of children without SHS exposure, which may be for the same reason.

Current reports indicate that smoke-free legislation has led to an increase of total home smoking bans [27–30]. Scotland has achieved a 50% reduction in the number of children exposed to SHS at home in the past 4 years (from 12% to 6%) through governmental policy and national targeting of this issue using the www.rightoutside.org approach and a comprehensive media education campaign. However, it is unlikely that the increased rates of home smoking bans have limited the recruitment of eligible families for the PREPASE study, as the study was able to identify sufficient numbers of eligible families.

As mentioned previously, SHS exposure in children is particularly prevalent in the group of socially deprived families [2, 31–33]. But the findings of this current study imply that there is possibly also a hidden group of highly educated parents who expose their children to SHS and are not willing to quit either. The majority of the responders to our questionnaires reported to have middle-academic education and just a limited amount of parents reported to have a low education. Maybe the recruitment strategies did not reach this group effectively. Still, the questionnaires and study information were all provided in layman language. The questionnaire via schools was also shorter and computerized compared to the questionnaire via the communities and physicians, but this did not result in higher response rates. We identified a lack of knowledge of what constitutes SHS exposure. Many parents believe that they don’t expose their child to SHS when they actually do. They believe that only smoking in one room, or smoking at the back door or with windows open mean their child isn’t exposed. There clearly is a need for education by means of a media campaign.

### Comparison with other studies

Literature concerning the recruitment of participants for similar studies is limited. Our results are comparable with the results of the REFRESH study in terms of recruitment [21]. This intervention study used 23 primary care practices in Scotland and recruited 68 mothers from 1,693 that were identified as smokers who had a child <5 yrs. of age living at home. Similar problems about the accuracy of primary care patient lists/details were mentioned in that study. Klosky et al. studied factors that influence non-participation in an intervention study to reduce SHS exposure in children diagnosed with cancer [34]. Barriers to participate included parental smoking status, parental gender, and child’s health. Smoking parents were less likely to enrol in the study than non-smoking parents, according to the authors possibly due to guilt or the burden associated with changing their smoking behaviour.

### Strength and limitations

The strength of this study is that valuable lessons have been learned from the recruitment process of the PREPASE study that provides insight into the problem of preventing SHS exposure in children. However, there are some limitations. First, we do not have additional information of all the physicians who were invited to recruit participants for the PREPASE study. Only 4 replied concerning possible participants, but that the rest did not reply does not necessarily mean that they did not try to recruit participants for the study. Likewise, the availability of characteristics of the non-participating parents were also limited and the actual number of parents exposed to one or more of the recruitment strategies are unknown. These limitations are mainly due to some ethical restrictions. Participation in the study was voluntary and parents could receive up to one reminder letter, as has been decided by the Medical Ethical Committee. More extensive analysis of the non-participants could have been performed by means of a qualitative study. But this was not possible in the time-span of the project due to the study delay resulting from the extensive recruitment process. Although the characteristics between the responders and non-responders (questionnaires A and B) were equal, it is still probable that selective participation has occurred. Parents of children with SHS exposure could have been less motivated to participate with one of the recruitment strategies compared to parents of children without SHS. Furthermore, misclassification could also be a problem due to parental underreporting of their smoking behaviour. Before the distribution of questionnaires, we submitted all questionnaires and information leaflets to a group of parents/families (*n* = 10) with different levels of education. The suggestions and points for improvement identified by this group were used to improve the questionnaires and leaflets. However, we cannot exclude that the questionnaire was still not able to reach the target population adequately. The number of mothers/fathers exposing the child to SHS did differ between families participating and not participating in the trial. Finally, the observations in this study may not be generalizable to other countries.

### Implications

The public health impact of successful interventions is dependent on its reach on population level. From this current study it is clear that reaching parents who expose their children to SHS is not effortless. However, if physicians were actively involved, the response of families was higher. Therefore, it is important that physicians are motivated to participate more actively in tackling the problem of SHS exposure in children and research on this topic. This study indicated a need for training on how physicians and health care professionals engage with smoking parents. The REFRESH study developed a How-To-Guide for practitioners. It is important that that policy makers and practitioners give priority to the protection of children from SHS exposure [21]. So far, the cooperation of physicians involved in care to children of this age to contribute to this project was limited. Additionally, the low participation of physicians in this study suggests that they may not address SHS exposure in children during consultations as frequently as required. Therefore, their practices regarding prevention of SHS exposure in children should be evaluated. More research should be done on how to overcome parental resistance due to for instance feelings of guilt/shame regarding SHS exposure in children, so that they can be reached effectively, not only for research but also for help for stopping SHS exposure in their children. Possibly, parents might be more comfortable with an electronic internet based program aimed at educating parents about SHS and how to stop SHS exposure in children.

## Conclusions

The attempted recruitment strategies did not result in effective recruitment of participants for the PREPASE study, possibly due to the subject “SHS exposure in children” and other factors influencing parental participation. The presence of asthma in the family, respiratory symptoms in the children and even incentives did not increase parental motivation for participation. When the responsible physicians participated, the motivation of parents to participate increased. However, an important observation during the recruitment process of the study is the limited contribution of the physicians working in child care settings.

## Additional file

Additional file 1: Questionnaires specifically developed for the PREPASE study. (DOCX 69 kb)

## Abbreviations

95% CI: 95% Confidence Interval
ADEM: Asthma DEtection and Monitoring
ISAAC: International Study of Asthma and Allergies in Childhood
MIKADO: Dutch acronym for Multifactorial intervention for children with asthma and overweight
MUMC: Maastricht University Medical Centre
OR: Odds Ratio
PREPASE: PREvention of PAssive Smoke Exposure
RCT: Randomized Controlled Trial
RNH: Dutch acronym for Registration Network of Family Physicians
SHS: Second-Hand Smoke
SPSS: Statistical Package for the Social Sciences
YHC: Youth Health Care
