# Supplementary material for: Recruiting families for an intervention study to prevent second-hand smoke exposure in children
Source: BMC Pediatr. 2018 Jan 31;18:19. doi: 10.1186/s12887-018-0983-4 (PMC5793411; doi:10.1186/s12887-018-0983-4)
Supplement: Additional file 1: — Questionnaires specifically developed for the PREPASE study. (DOCX 69 kb) [file 12887_2018_983_MOESM1_ESM.docx]

**Additional file 1: questionnaires specifically developed for the PREPASE study**

**QUESTIONNAIRE A**

**General questions**

1. Date of completion: _ _ - _ _ - 20_ _ (dd/mm/yyyy)

2. Child birth date: _ _ - _ _ - _ _ _ _ (dd/mm/yyyy)

| - Male | - Female |
| --- | --- |

3. What is the sex of your child?

4. What is the relationship of you and your partner to your child?

|  | **Yourself** | **Your partner** |
| --- | --- | --- |
| Biological mother |  |  |
| Biological father |  |  |
| Stepmother |  |  |
| Stepfather |  |  |
| Other, namely: | - …………………………. | - …………………………. |
| Not applicable |  | - *(I do not have a partner)* |

5. What are the birth dates of you and your partner?

Yourself Your partner

_ _ - _ _ - _ _ _ _ (dd/mm/yyyy) _ _ - _ _ - _ _ _ _ (dd/mm/yyyy)

- Not applicable *(I do not have a partner)*

6. a. Do you have more children?

| - Yes | - No 🡪 if ‘No’ go to question 7 |
| --- | --- |

b. Record for every other child their birth date. Also specify if this child is the biological brother/sister of the child mentioned at question 2 and whether he/she lives at home. *(With biologically we mean from the same father and mother.)*

| **Birth date *(dd/mm/yyyy)*** | **Relation brother/sister** | **Lives at home?** |
| --- | --- | --- |
| _ _ - _ _ - _ _ _ _ | - Biological - Half - Other | - Yes - No   Etc. |

1. Which situation does currently apply for you and your partner? *More answers are possible.*

|  | **Yourself** | **Your partner** |
| --- | --- | --- |
| Working outdoors, 20 hours/more per week |  |  |
| Working indoors, less than 20 hours per week |  |  |
| Looking for work |  |  |
| Incapacitated |  |  |
| Working indoors (household) |  |  |
| Studying |  |  |
| Other, namely: | - ………………… | - *…………………* |
| Not applicable |  | - *(I do not have a partner)* |

1. What is your highest level of education? And your partners’?

|  | **Yourself** | **Your partner** |
| --- | --- | --- |
| Primary school |  |  |
| Lower vocational education |  |  |
| General secondary education |  |  |
| Middle vocational education |  |  |
| Senior general secondary education, pre-university |  |  |
| Higher vocational education |  |  |
| University, academic |  |  |
| Other, namely: | - ………………… | - ………………… |
| Not applicable |  | - *(I do not have a partner)* |

1. In which country were you and your partner born?

|  | **Yourself** | **Your partner** |
| --- | --- | --- |
| Netherlands |  |  |
| Suriname |  |  |
| Turkey |  |  |
| Morocco |  |  |
| Netherlands Antilles |  |  |
| Other, namely: | - ………………… | - ………………… |
| Not applicable |  | - *(I do not have a partner)* |

**Questions 10 – 47 are about the health of your child**

*(Questions 10 - 16: Questionnaire on Eczema (Module 1.4 obtained from the International Study of Asthma and Allergies in Childhood (ISAAC) phase II [*[*1*](#_ENREF_1)*])*

1. Has your child ever had an itchy rash which was coming and going for at least six months?

| - Yes | - No 🡪 if ‘No’ go to question 16 |
| --- | --- |

11. Has your child had this itchy rash at any time in the last 12 months?

| - Yes | - No 🡪 if ‘No’ go to question 16 |
| --- | --- |

12. Has this itchy rash at any time affected any of the following places: the folds of the elbows, behind the knees, in front of the ankles, under the buttocks, or around the neck, ears or eyes?

| - Yes | - No |
| --- | --- |

1. At what age did this itchy rash first occur?

| - - Under 2 years   - Age 2-4 years   - Age 5 or more |
| --- |

1. Has this rash cleared completely at any time during the last 12 months?

| - Yes | - No |
| --- | --- |

1. In the last 12 months, how often, on average, has your child been kept awake at night by this itchy rash?

| - Never - Less than one night per week - One or more nights per week |
| --- |

1. Has your child ever had eczema?

| - Yes | - No |
| --- | --- |

*(Questions 17, 19 - 23, 26: Questionnaire on Wheezing (Module 1.2 ISAAC phase II [*[*1*](#_ENREF_1)*]))*

17. Has your child ever had wheezing or whistling in the chest at any time in the past?

| - Yes | - No 🡪 if ‘No’ go to question 23 |
| --- | --- |

18. In which period of his/her life has your child had wheezing or whistling in the chest? *More answers are possible.*

| - In the first year of life - In the second year of life - In the third year of life - In the fourth year of life - In the fifth year of life - Other, namely ……………. |
| --- |

19. In the last 12 months, has your child had wheezing or whistling in the chest?

| - Yes | - No 🡪 if ‘No’ go to question 23 |
| --- | --- |

20. How many attacks of wheezing has your child had in the last 12 months?

| - None - 1-3 - 4-12 - More than 12 |
| --- |

21. In the last 12 months, how often, on average, has you child’s sleep been disturbed due to wheezing?

| - Never - Less than one night per week - One or more nights per week |
| --- |

22. In the last 12 months, has wheezing ever been severe enough to limit your child’s speech to only one or two words at a time between breaths?

| - Yes | - No |
| --- | --- |

23. In the last 12 months, has your child’s chest sounded wheezy during or after exercise?

| - Yes | - No |
| --- | --- |

24. Has your child ever had shortness of breath?

| - Yes | - No 🡪 if ‘No’ go to question 26 |
| --- | --- |

25. Has your child ever had shortness of breath in the past 12 months?

| - Yes | - No |
| --- | --- |

26. Has your child ever had asthma (physician diagnosed)?

| - Yes | - No |
| --- | --- |

*(Question 27: Asthma management (Module 2.2 ISAAC phase II [*[*1*](#_ENREF_1)*]))*

27. a. In the past 12 months, has your child used any medicines, pills, puffers, or other medication for wheezing or asthma?

| - Yes | - No 🡪 if ‘No’ go to question 28 |
| --- | --- |

b. Please name the medication(s)

| **Medicine** | **How often? (please circle one or both)** | |
| --- | --- | --- |
| ……………… | When wheezy | Regularly (every day for at least two months of the year) |
| ……………… | When wheezy | Regularly (every day for at least two months of the year) |

*(Question 28: Questionnaire on Wheezing (Module 1.2 ISAAC phase II [*[*1*](#_ENREF_1)*]))*

28. In the last 12 months, has your child had a dry cough at night, apart from a cough associated with a cold or chest infection?

| - Yes | - No 🡪 if ‘No’ go to question 30 |
| --- | --- |

29. In the last 12 months, how often, on average, has you child’s sleep been disturbed due to coughing at night, apart from cough associated with a cold or chest infection?

| - Never - Less than one night per week - One or more nights per week |
| --- |

*(Questions 30-35: Questionnaire on rhinitis (Module 1.3 ISAAC phase II [*[*1*](#_ENREF_1)*]))*

30. Has your child ever had a problem with sneezing or a runny or blocked nose, when he/she did not have a cold or the flu?

| - Yes | - No 🡪 if ‘No’ go to question 35 |
| --- | --- |

31. In the past 12 months, has your child had a problem with sneezing or a runny or blocked nose when he/she did not have a cold or the flu?

| - Yes | - No 🡪 if ‘No’ go to question 35 |
| --- | --- |

32. In the past 12 months, has this nose problem been accompanied by itchy-watery eyes?

| - Yes | - No |
| --- | --- |

33. In which of the past 12 months did this nose problem occur*? More answers are possible.*

| - January | - May | - September |
| --- | --- | --- |
| - February | - June | - October |
| - March | - July | - November |
| - April | - august | - December |

34. In the past 12 months, how much did this nose problem interfere with your child’s daily activities?

| - Not at all - A little - A moderate amount - A lot |
| --- |

35. Has your child ever had hay fever?

| - Yes | - No |
| --- | --- |

36. In the past 12 months, has your child been diagnosed by a physician with one or more of the following health problems? If yes, has your child received prescribed medications and was he/she admitted in the hospital because of the health problem?

Please give an answer for each of the following health problems:

|  | **Physician diagnosed?** | | **Prescribed medication?** | | **Admitted in the hospital?** | |
| --- | --- | --- | --- | --- | --- | --- |
| Hay fever | - Yes | - No | - Yes | - No | - Yes | - No |
| Flu or severe cold | - Yes | - No | - Yes | - No | - Yes | - No |
| Throat infection | - Yes | - No | - Yes | - No | - Yes | - No |
| Middle ear infection | - Yes | - No | - Yes | - No | - Yes | - No |
| Sinusitis | - Yes | - No | - Yes | - No | - Yes | - No |
| Bronchitis | - Yes | - No | - Yes | - No | - Yes | - No |
| Pneumonia | - Yes | - No | - Yes | - No | - Yes | - No |

37. Did your child ever had other respiratory tract infections apart from those mentioned in question 36?

| - Yes, namely ……………. - No |
| --- |

38. In the past 12 months, how often did your child had a respiratory tract infection (such as, flu, severe cold, throat infections, middle ear infection, sinusitis, bronchitis or pneumonia) whereby you had to consult a physician?

| - Never - 1-2 times - 3-5 times - 6 times or more |
| --- |

39. a. Does your child get vitamin supplements?

| - Yes | - No 🡪 if ‘No’ go to question 40 |
| --- | --- |

b. Which vitamin supplements does your child get? Is this daily, occasionally or never?

|  | **Daily** | **Occasionally** | **Never** |
| --- | --- | --- | --- |
| Vitamin A |  |  |  |
| Vitamin B |  |  |  |
| Vitamin C |  |  |  |
| Vitamin D |  |  |  |
| Vitamin E |  |  |  |
| Omega 3 |  |  |  |
| Other:………… |  |  |  |

40. a. At how many weeks pregnancy was your child born? _ _ weeks

b. What was the birth weight of your child? _ _ _ _ grams

41. a. Did your child suffered from any health problems while your were pregnant with or gave birth to him/her?

| - Yes | - No 🡪 if ‘No’ go to question 42 |
| --- | --- |

b. Which health problems did your child suffered while you were pregnant with or gave birth to him/her? *More answers are possible.*

| - Growth retardation during pregnancy - Infections - Lack of oxygen - Other: …………………………………… |
| --- |

42. a. Was your child ever breastfed?

| - Yes: - Less than 6 months, namely _ _ weeks - 6 -12 months - More than 12 months | - No 🡪 if ‘No’ go to question 43 |
| --- | --- |

b. For how long was your child breastfed without adding other foods or juices?

| - Less than 2 months - 2 - 4 months - 5 - 6 months - More than 6 months |
| --- |

43. Did you or your partner smoke during or after the pregnancy of your child?

|  | **Yourself** | **Your partner** |
| --- | --- | --- |
| **During the pregnancy** | - Yes | - Yes |
|  | - No | - No |
|  |  |  |
| **After the pregnancy** | - Yes | - Yes |
|  | - No | - No |

44. a. Did a physician ever diagnose an inherent abnormality of the hart and/or lungs of your child?

| - Yes | - No 🡪 if ‘No’ go to question 45a |
| --- | --- |

b. Which inherent abnormality of the hart and/or lungs of your child has been diagnosed? ………………………………

45. a. Has your child been diagnosed with a syndrome by a physician?

| - Yes | - No 🡪 if ‘No’ go to question 46 |
| --- | --- |

b. Which syndrome has been diagnosed in your child? ………………………………

46. a. During the ages of 0 through 4 years, did your child ever go to a form of child care facility?

| - Yes | - No 🡪 if ‘No’ go to question 47 |
| --- | --- |

b. Which type of child care facility did your child attend?

| - Grandparent(s) - Host parents - Day care, nursery - Other: …………………………………… |
| --- |

47. For each health complaint below, please specify if your child has a biological relative with one of these complaints. Also, indicate whether this has been diagnosed by a physician.

| **Asthma family member?** (more answers are possible) | **Diagnosed by a physician?** | |
| --- | --- | --- |
| - No family member - Father - Mother - Biological sibling(s) - Half sibling(s) - Other:……………….. | - Yes - Yes - Yes - Yes - Yes | - No - No - No - No - No |
| **Eczema family member?** (more answers are possible) | **Diagnosed by a physician?** | |
| - No family member - Father - Mother - Biological sibling(s) - Half sibling(s) - Other:……………….. | - Yes - Yes - Yes - Yes - Yes | - No - No - No - No - No |
| **Hay fever family member?** (more answers are possible) | **Diagnosed by a physician?** | |
| - No family member - Father - Mother - Biological sibling(s) - Half sibling(s) - Other:……………….. | - Yes - Yes - Yes - Yes - Yes | - No - No - No - No - No |

**The following questions refer to your smoking habits**

*(Questions 48-61: Measurement instrument for research on smoking and smoking cessation [*[*2*](#_ENREF_2)*])*

1. Do you sometimes smoke?

| - Yes, daily 🡪 go to question 53 - Yes, sometimes 🡪 go to question 53 - No, I am taking a quit attempt now 🡪 go to question 49 - No, not at all, or I stopped smoking more than 6 months ago 🡪 go to question 66 |
| --- |

1. How long has it been that you stopped smoking?

| - Less than 1 week, namely _ days - Less than 1 month, namely _ weeks - More than 1 month, but less than 6 months, namely _ months |
| --- |

1. Have you smoked since you stopped smoking?

| - No, not one puff - Yes, 1-5 cigarettes - Yes, more than 5 cigarettes |
| --- |

1. Did you persevere not to smoke for 24 hours or more at this stop attempt?

| - Yes, _ _ times - No |
| --- |

1. Have you used any aid or methods to stop smoking since you quit smoking?

More answers are possible.

| - No, no aid used - No-smoking course or group therapy - Nicotine gum - Nicotine band-aid - Nicotine pastilles - Nicotine microtabs (tablet for under the tong) - Zyban (bupropion) - Speaking to GP about smoking cessation - Acupuncture - Laser therapy - Telephone helpline - Folder - Book - Other: …………………………………. |
| --- |

**Here are a few questions about your smoking habit. If you are presently smoking, the following questions are related to your current smoking habit. If you are currently attempting to quit smoking, the following questions are related to the period when you were smoking.**

1. Which and how many of the following tobacco products do you smoke on average per day?

More answers are possible.

| - _ _ cigarettes per day - _ _ roll-ups per day - _ _ cigars/cigarillo’s per day - _ _ pipe per day |
| --- |

1. Have you smoked one or more cigarettes (roll-ups, cigars, pipe) over the past 24 hours?

| - Yes | - No |
| --- | --- |

1. Have you smoked one or more cigarettes (roll-ups, cigars, pipe) over the past 7 days?

| - Yes | - No |
| --- | --- |

*(Question 56: the Transtheoretical Model of Change [*[*3*](#_ENREF_3)*])*

1. Are you planning to stop smoking in the future?

| - Not applicable, I am attempting to quit now - Yes, within 1 month - Yes, within 6 months, not the coming month - Yes within 1 year, , but not the coming 6 months - Yes, within 5 years - Yes, but not within 5 years - No, not planning to quit |
| --- |

*(Question 57 -61: Fagerstöm Test for Nicotine Dependence [*[*4*](#_ENREF_4)*])*

1. How soon after you wake up do you start smoking your first cigarette?

| - Within 5 minutes - 6-30 minutes - 31-60 minutes - After 60 minutes |
| --- |

1. Do you find it difficult to refrain from smoking in places where it is forbidden (e.g. cinema, school, hospital, public transportation)?

| - Yes | - No |
| --- | --- |

1. Which cigarette would you hate most to give up?

| - The first one in the morning - All others (doesn’t matter which one) |
| --- |

1. Do you smoke more frequently during the first hours after waking than during the rest of the day?

| - Yes | - No |
| --- | --- |

1. Do you smoke if you are so ill that you are in bed most of the day?

| - Yes | - No |
| --- | --- |

1. Do you smoke inside your house?

| - Yes 🡪 if “Yes” go to question 63a - No 🡪 if “No” go to question 64a |
| --- |

1. a. Where do you smoke inside your house?

*More answers are possible.*

| - Living room - Your own bedroom - Kids bedroom - Kitchen - Under the cooker hood - Hallway - Dining room - Attic - Restroom - Other rooms, namely: …………………………………. |
| --- |

b. Do you open a window or door when you smoke inside your house?

| - Yes | - No |
| --- | --- |

1. a. Where do you smoke outside your house?

*More answers are possible.*

| - Balcony - Yard - Car - Other, namely: …………………………………. |
| --- |

b. Do you open the window when smoking in the car?

| - Yes - No - Not applicable (because I don’t smoke in the car) |
| --- |

1. a. Do you smoke in the presence of your child?

| - Yes - No 🡪 if “No” go to question 66 |
| --- |

b. Where do you smoke in the presence of your child and does this happen always, often, occasionally, or never?

*Please give an answer for each situation.*

|  | **Always** | **Often** | **Occasionally** | **Never** |
| --- | --- | --- | --- | --- |
| Living room |  |  |  |  |
| Own bedroom |  |  |  |  |
| Kids bedroom |  |  |  |  |
| Kitchen |  |  |  |  |
| Cooker hood |  |  |  |  |
| Dining room |  |  |  |  |
| Hallway |  |  |  |  |
| Attic |  |  |  |  |
| Toilet/bathroom |  |  |  |  |
| Balcony |  |  |  |  |
| Yard |  |  |  |  |
| Car |  |  |  |  |
| Other: ……… |  |  |  |  |

c. Are you willing to take measures to prevent tobacco smoke exposure to your child?

| - Yes | - No 🡪 if ‘No’ go to question 66 |
| --- | --- |

d. Which difficulties do you expect when taking measures to prevent tobacco smoke exposure to your child? *More answers are possible.*

I expect…

| - no difficulties - to find it difficult for myself to not smoke inside the house - to find it difficult with the smoking of partner - to find it difficult with the smoking of families and/or visitors - to find it difficult with judgment or lack of understanding from family members and/or visitors - to find it difficult with other things, namely: ………………………………. |
| --- |

**The following questions are about the smoking habits of your partner. If you do not have a partner, please fill this in at question 66.**

**Partner: your current partner (not necessarily the biological father/mother of your child).**

1. Does your partner sometimes smoke?

| - Yes, daily 🡪 go to question 71 - Yes, sometimes 🡪 go to question 71 - No, he/she is taking a quit attempt now 🡪 go to question 67 - No, not at all, or he/she stopped smoking more than 6 months ago 🡪 go to question 84 - I do not have a partner 🡪 go to question 84 |
| --- |

*(Questions 67-83 are the same as questions 49-65, but referring to the smoking behavior of the partner.)*

**Additional general questions about smoking**

84. In which areas of the house is smoking not allowed? *More answers are possible.*

| - All - Living room - Bedroom partners - Bedroom child - Kitchen - Dinner table / room - Hallway - Attic - Toilet - Other, namely: …………………………………. |
| --- |

85. a. Apart from you or your partner, do other people smoke inside your house?

| - Yes | - No 🡪 if ‘No’ go to question 86 |
| --- | --- |

b. Apart from you or your partner, which other people smokes in your house? Can you give an indication on how frequent this occurs? *Please provide an answer for each category.*

|  | Daily | 4-6 times per wk | 1-3 times per wk | 1-2 times per wk | Occasionally (max. 3 times per month) | Never |
| --- | --- | --- | --- | --- | --- | --- |
| Grandparent(s) |  |  |  |  |  |  |
| Friends |  |  |  |  |  |  |
| Sitter |  |  |  |  |  |  |
| Family |  |  |  |  |  |  |
| Neighbor |  |  |  |  |  |  |
| Other child (sibling(s)) |  |  |  |  |  |  |
| Others:…….. |  |  |  |  |  |  |

86. On average, how many times is your child present in a room where others are smoking at that moment?

| - Never - Occasionally (max. 3 times per month) - 1-2 times per month - 1-3 times per week - 4-6 times per week - Every day |
| --- |

87. On average, how many times is your child present in a room where people have smoked, when your child was not present at that moment?

| - Never - Occasionally (max. 3 times per month) - 1-2 times per month - 1-3 times per week - 4-6 times per week - Every day |
| --- |

**“Since July 2008, smoking is prohibited in public areas in the Netherlands.”**

88. Do you think that this measure is a good idea?

| - Yes | - No |
| --- | --- |

89. How has this affected you? ……………………………………………………….

90. Are you aware that your surroundings are also smoking with you when you are smoking at the moment?

| - Yes | - No 🡪 END questionnaire |
| --- | --- |

91. Where/how did you receive information about passive smoking? *More answers are possible.*

| - Internet - STIVORO - General practitioner - School - Other, namely:……………………………………. |
| --- |

**END of the Questionnaire**

References:

1. **International Study of Asthma and Allergies in Childhood. Phase II Modules**.

2. Mudde A.N. WMC, Kremers S., Vries de H.: **Meetinstrumenten voor onderzoek naar roken en stoppen met roken. *(Measurement instruments for research on smoking and smoking cessation)***, Tweede druk *(second edition)* edn. Den Haag, Nederland *(The Hague, Netherlands)*: STIVORO voor een rookvrije toekomst *(STIVORO for a smokefree future)*; 2006.

3. Fava JL, Velicer WF, Prochaska JO: **Applying the transtheoretical model to a representative sample of smokers**. *Addictive behaviors* 1995, **20**(2):189-203.

4. Heatherton TF, Kozlowski LT, Frecker RC, Fagerstrom KO: **The Fagerstrom Test for Nicotine Dependence: a revision of the Fagerstrom Tolerance Questionnaire**. *British journal of addiction* 1991, **86**(9):1119-1127.

**Questionnaire B**

1. Date of completion: _ _ - _ _ - 20_ _ (dd/mm/yyyy)

2. Child birth date: _ _ - _ _ - _ _ _ _ (dd/mm/yyyy)

3. What is the relationship of you and your partner to your child?

|  | **Yourself** | **Your partner** |
| --- | --- | --- |
| Biological mother |  |  |
| Biological father |  |  |
| Stepmother |  |  |
| Stepfather |  |  |
| Other, namely: | - …………………………. | - …………………………. |
| Not applicable |  | - (I do not have a partner) |

1. Do you and/or your partner smoke and does this happens in the presence of your child?

|  | **Current smoking** | **Smoking in the presence of your child** |
| --- | --- | --- |
| You | - Yes - No | - Yes - No |
|  |  |  |
| Partner | - Yes - No | - Yes - No |

1. Are you planning to stop smoking in the future?

| - Not applicable, I am attempting to quit now - Yes, within 1 month - Yes, within 6 months, not the coming month - Yes within 1 year, , but not the coming 6 months - Yes, within 5 years - Yes, but not within 5 years - No, not planning to quit |
| --- |

6. Where do you and/or your partner smoke in the presence of your child and does this happen always, often, occasionally, or never?

*Please give an answer for each situation.*

|  | **Always** | **Often** | **Occasionally** | **Never** |
| --- | --- | --- | --- | --- |
| Living room |  |  |  |  |
| Own bedroom |  |  |  |  |
| Kids bedroom |  |  |  |  |
| Kitchen |  |  |  |  |
| Cooker hood |  |  |  |  |
| Dining room |  |  |  |  |
| Hallway |  |  |  |  |
| Attic |  |  |  |  |
| Toilet/bathroom |  |  |  |  |
| Balcony |  |  |  |  |
| Yard |  |  |  |  |
| Car |  |  |  |  |
| Other: ……… |  |  |  |  |

7. Has your child ever had asthma (physician diagnosed)?

| - Yes | - No |
| --- | --- |

8. Has a biological parent or sibling of your child been diagnosed with asthma?

| - Yes, namely ……………………………………… - No |
| --- |

9. In the past 12 months, how often did your child had a respiratory tract infection (such as, flu, severe cold, throat infections, middle ear infection, sinusitis, bronchitis or pneumonia) whereby you had to consult a physician?

| - Never - 1-2 times - 3-5 times - 6 times or more |
| --- |

10. In the last 12 months, has your child had wheezing or whistling in the chest?

| - Yes | - No |
| --- | --- |

11. How many attacks of wheezing has your child had in the last 12 months?

| - None - 1-3 - 4-12 - More than 12 |
| --- |

12. What is your reason(s) for not participating in the study? *More answers are possible.*

| - No interest - Lack of time - You are currently moving - Your child does not have respiratory complaints - Your child has a lot of respiratory complaints, and the burden is heavy - Smoking is not allowed in your house - Your child has been exposed to tobacco smoke for years, and has never experienced complaints because of the tobacco smoke exposure - You have no reason / you do not want to give a reason - Other, namely:………………………………………………….. |
| --- |

**Questionnaire for Schools**

**General questions**

*The following questions from questionnaire A are asked: questions 1-4, 6a, 7-9.*

*Additionally the following questions are asked:*

- What is the first name of your child? …………………………

What is the last name of your child? …………………………

- What is the name of the school of your child? …………………………

In which group is your child? .

**Questions about the child’s health**

*The following questions from questionnaire A are asked: questions 17-29, 36-38, 40-45, 47*

**Questions about parental smoking habits**

*The following questions from questionnaire A are asked: questions 48, 53, 62, 65a (for both parents).*
